# Different Heat Shock Proteins Bind α-Synuclein With Distinct Mechanisms and Synergistically Prevent Its Amyloid Aggregation

**DOI:** 10.3389/fnins.2019.01124

**Published:** 2019-11-01

**Authors:** Chunyu Jia, Xiaojuan Ma, Zhenying Liu, Jinge Gu, Xiang Zhang, Dan Li, Shengnan Zhang

**Affiliations:** ^1^Interdisciplinary Research Center on Biology and Chemistry, Shanghai Institute of Organic Chemistry, Chinese Academy of Sciences, Shanghai, China; ^2^Interdisciplinary Research Center on Biology and Chemistry, Shanghai Institute of Organic Chemistry, University of the Chinese Academy of Sciences, Beijing, China; ^3^Key Laboratory for the Genetics of Developmental and Neuropsychiatric Disorders (Ministry of Education), Bio-X Institutes, Shanghai Jiao Tong University, Shanghai, China

**Keywords:** Parkinson’s disease, α-syn, amyloid aggregation, heat shock protein, protein homeostasis, synergistic effect

## Abstract

α-Synuclein (α-Syn) forms pathological amyloid aggregates deposited in Lewy bodies and Lewy neurites in the brain of Parkinson’s disease (PD) patients. Heat shock proteins (Hsps) are the major components of the cellular chaperone network, which are responsible for preventing proteins from amyloid aggregation. Different Hsps were reported to interact with α-syn. However, the underlying mechanism of the interplay between α-syn and different Hsps remains unclear. Here, by combing NMR spectroscopy, electron microscope and other biochemical approaches, we systemically investigated the interaction between α-syn and three Hsps from different families including Hsp27, HDJ1, and Hsp104. We found that all three Hsps can weakly bind to α-syn and inhibit it from amyloid aggregation. Intriguingly, different Hsps recognize distinct regions of α-syn monomer, and act synergistically in chaperoning α-syn from fibril formation in sub-stoichiometry. Our results revealed the diverse binding mechanisms employed by different Hsps to tackle α-syn, and suggested that different Hsps form a network for cooperatively chaperoning α-syn from pathological aggregation.

## Introduction

Maintenance of protein homeostasis is essential in living cells (Gidalevitz et al., 2011; Balchin et al., 2016), failure of which may lead to abnormal protein aggregation that closely associated with a variety of devastating human neurodegenerative disease including Alzheimer’s disease, Parkinson’s disease (PD), and amyotrophic lateral sclerosis (Chiti and Dobson, 2006; Eisenberg and Jucker, 2012; Cohen et al., 2015; Yerbury et al., 2016). Molecular chaperone represents the major class of proteins that are responsible for maintaining the cellular protein homeostasis (Powers et al., 2009; Hartl et al., 2011; Brehme et al., 2014). There are over 180 molecular chaperones in human (Freilich et al., 2018a). Among them, the most important and well-studied family belongs to the heat shock protein (Hsp) family (Hartl et al., 2011). The family members include Hsp100, Hsp90, Hsp70, Hsp60, Hsp40, and the small Hsps (sHsps), which are classified by their distinct molecular mass in the unit of kilodalton (Saibil, 2013). They commonly exist with high abundancy throughout the three kingdoms of life. Different members of Hsp family feature distinct chaperone activities (e.g., disaggregase activity of Hsp100, holdase activity of Hsp40 and sHsps, and foldase activity of Hsp60), and form an elaborate network for maintaining the protein homeostasis (Itoh et al., 1995; Glover and Lindquist, 1998; Shorter and Lindquist, 2004; Hasegawa et al., 2017; Liu et al., 2017, 2018).

α-Synuclein (α-Syn) is a highly abundant protein in human brain (Kalia and Kalia, 2015). Under disease condition where protein homeostasis is severely disturbed, α-syn forms pathological amyloid aggregates that is believed to be causative to PD (Jellinger, 2003; Chiti and Dobson, 2006; Goedert et al., 2013). Moreover, deposition of α-syn amyloid aggregates in the brains of patients represents the pathological hallmarks of PD and other synucleinopathies (Crowther et al., 2000; Hansen et al., 2011). α-Syn is composed of 140 amino acids that can be divided into three regions: an N-terminal lipid-binding region, a central non-amyloid-β component (NAC) region that forms the fibril core, and a C-terminal acidic region (Lashuel et al., 2013; Tuttle et al., 2016; Wang et al., 2016; Tian et al., 2019). Despite that α-syn is highly prone to aggregate into amyloid fibrils by itself *in vitro* and under disease condition *in vivo* (Hansen et al., 2011; Volpicelli-Daley et al., 2011; Guerrero-Ferreira et al., 2018; Li B. et al., 2018; Li Y. et al., 2018), endogenous α-syn resists to aggregate in the normal intracellular environment. Molecular chaperones are believed to play an essential role in maintaining the physiological configuration of endogenous α-syn and preventing it from aggregation (Auluck et al., 2002). Indeed, different members of Hsp family have been reported to be closely associated with α-syn aggregation both *in vitro* and in the brains of PD patients (Kong et al., 2005; Lo Bianco et al., 2008).

A large number of Hsps, such as Hsp90, Hsp70, Hsp60, Hsp40, and sHsps (Hsp27 and HspB5), have been identified to co-precipitate with α-syn in the Lewy bodies and Lewy neurites in PD patients (McLean et al., 2002; Bruinsma et al., 2011; Pemberton et al., 2011; Cox et al., 2018). The expression level of Hsp27 is dramatically up-regulated in the patient brains of synucleinopathies (Outeiro et al., 2006). Moreover, Hsp27 can prevent α-syn aggregation *in vitro*, and reduce α-syn cytotoxicity in cell models (Zourlidou et al., 2004; Cox et al., 2018). Hsp40, the well-known co-chaperone of Hsp70 (Michels et al., 1997), was found to be able to slow down the aggregation of α-syn *in vitro* (Hasegawa et al., 2017). In addition, Hsp70 exhibits chaperone activity to prevent α-syn fibril formation both *in vitro* and *in vivo* (Pemberton et al., 2011; Gao et al., 2015). The Hsp100 family can disaggregate preformed α-syn fibrils and attenuate the cytotoxicity of α-syn aggregates in the fly and yeast models (Schirmer et al., 1996; Weber-Ban et al., 1999; Lo Bianco et al., 2008). Although different Hsps were found to prevent α-syn aggregation, the molecular basis underlying the recognition between α-syn and different Hsps remains unclear. Nor do we know whether they could synergistically modulate α-syn aggregation.

In this study, we systemically investigate the interplay between α-syn and three different Hsps including Hsp27 from sHsps, HDJ1 from Hsp40, and Hsp104 from Hsp100 family. By combining NMR spectroscopy, electron microscope (EM) and other biochemical approaches, we found that all these three Hsps bind to α-syn with the binding affinity in a millimolar range, and effectively inhibit α-syn fibril formation. Interestingly, different Hsps recognize distinct regions of α-syn monomer. For instance, HDJ1 mainly interacts with the C-terminal of α-syn by its C-terminal domain. Whereas, Hsp27 utilizes its core α-crystallin domain to interact with the N-terminal of α-syn. Hsp104 binds to the N-terminal of α-syn by the nucleotide-binding domain 2. More importantly, we found that different Hsps may act synergistically in preventing α-syn aggregation. Our results imply that distinct Hsps may form a chaperone network to tackle α-syn in different yet complementary strategies and prevent it from pathological aggregation in a synergistic manner.

## Materials and Methods

### Plasmid Construction

Gene encoding Hsp27 was inserted into a pET-28a vector with an N-terminal His6-tag and a following tobacco etch virus (TEV) protease cleavage site. HDJ1 was constructed in a pPSET vector with an N-terminal His6-tag and a following thrombin protease cleavage site. Hsp104 was cloned into a pPROEX HTb vector with an N-terminal His6-tag and a following TEV protease cleavage site. Hsp27 α-crystallin domain (ACD, residues 85–176), HDJ1 C-terminal domain (CTD, residues 161–322), and Hsp104 nucleotide-binding domain 2 (NBD2, residues 556–870) were generated by polymerase chain reaction and subcloned. All constructions were confirmed by DNA sequencing (GENEWIZ, Inc., Suzhou, China).

### Protein Expression and Purification

All Hsps were expressed in *Escherichia coli* (*E. coli*) BL21(DE3) cells (Novagen) using the same expression and purification protocol. Cells were grown at 37°C to an approximate OD_600_ of 0.6, and induced by 500 μM IPTG. After shaking at 16°C for 12 h, the cells were harvested by centrifugation (5053 *g*, 16 min), and resuspended in the buffer of 50 mM Tris–HCl, 500 mM NaCl, 2 mM β-mercaptoethanol, 2 mM PMSF at pH 7.5, then lysed by a high-pressure homogenizer. Cell debris was removed by centrifugation (15,000 *g*, 45 min, 4°C), and the supernatant was loaded to a 5 mL HisTrapTM FF column (GE Healthcare). The column was then washed with five volumes of Ni buffer (50 mM Tris–HCl, 500 mM NaCl at pH 7.5). Proteins were eluted with Ni buffer plus 350 mM imidazole. The proteins were further purified by gel filtration using a Superdex 200 or 75 columns (GE Healthcare) in the buffer of 50 mM Na_2_HPO_4_, 150 mM NaCl, pH 7.0. The purified protein was concentrated and stored at −80°C. The purity was assessed by SDS-PAGE. Protein concentration was determined by BCA assay (Thermo Fisher).

Expression and purification of α-syn was the same as previously described (Liu et al., 2018). Briefly, α-syn was purified by a 5 mL HighTrap Q HP column (GE Healthcare), and followed by a Superdex 75 gel filtration column (GE Healthcare). ^15^N labeled proteins for NMR studies were grown in M9 minimal medium with ^15^N-NH_4_Cl (1 g/l) as the sole nitrogen source. The purification was the same as that for the unlabelled proteins.

### ThT Fluorescence Assay

ThT fluorescence assays were performed to monitor the aggregation of α-syn in the absence and presence of different Hsps. The ThT assays were conducted in a 384-well plate (black with flat optical bottom) in a Varioskan fluorescence plate reader (Thermo Scientific) with a ThT buffer containing 50 mM Tris–HCl, 150 mM NaCl at pH 7.0. Hsps were premixed with α-syn at ratios (α-syn/Hsps) of 1:50, 1:10, and 1:2, respectively. The samples of α-Syn in the presence of BSA at the same ratios (α-syn/BSA) of 1:50, 1:10, and 1:2 were made as negative controls. For the synergistic assays, the Hsps were premixed at equal molars and then mixed with α-syn at ratios (α-syn/Hsps) of 1:10, 1:50, and 1:200, respectively. A total volume of 60 μL premixed solution was added to each well. Samples were shaken using 600 rpm at 37°C and the fluorescence was measured with excitation at 440 nm and emission at 485 nm. The final concentrations in the reaction system were 100 μM α-syn, 50 μM ThT, 1% (w/w) α-syn fibril seeds (prepared by sonicating α-syn fibrils for 15 s), and varied concentrations of Hsps as indicated in the figures. Three repeats were performed for each experiment for statistical analysis, and at least three independent biological repeats were performed.

### Negative-Staining EM (NS-EM)

Directly after the ThT experiment, 7 μL of each sample was deposited onto a glow-discharged holy carbon EM grid covered with a thin layer of carbon film (Beijing Zhongjingkeyi Technology Co., Ltd) for 45 s, followed by washing in water (8 μL) twice. The grid was then stained by 3% (w/v) uranyl acetate for 45 s for staining. An FEI Tecnai T12 electron microscope operating at an accelerating voltage of 120 kV was used to examine and visualize the samples. Images were collected by a Gatan US4000 4k × 4k CCD camera.

### OCTET

The binding kinetics of Hsps and their variants to α-syn monomer were measured by Bio-layer interferometry (BLI) method on an ForteBio Octet RED96 system (Pall ForteBio LLC). Assays were performed at 30°C in a 96-well black flat bottom plate (Greiner Bio-One) with orbital shaking at 1,000 rpm in the assay buffer of 50 mM Tris–HCl, 150 mM NaCl at pH 7.0. A total volume of 200 μL solution was added to each well. Streptavidin biosensors (ForteBio) were incubated in the assay buffer for 1 min, then the biotinylated Hsp (25 μg/mL) were loaded onto the biosensors for 5 min, followed by the assay buffer for 2 min to remove unbound proteins. Then the association step was performed by incubating biosensors with different concentrations of α-syn monomer for 3 min and subsequently followed by a disassociation step with incubating with the assay buffer for 8 min. The resulting curves were corrected using the blank reference and analyzed by the ForteBio Data Analysis software 9.0.

### NMR Spectroscopy

All the NMR experiments were performed at 298 K on a Bruker 900 MHz or Agilent 800 MHz spectrometer with a cryogenic TXI in the NMR buffer of 25 mM Na_2_HPO_4_, 50 mM NaCl, and 10% (v/v) D_2_O at pH 7.0. Backbone resonance assignment of α-syn was accomplished according to the previous publication and verified by the collected 3D HNCA and HNCACB experiments (Liu et al., 2018). For titration assays, 25 μM ^15^N labeled α-syn was titrated by different Hsps at the molar ratio of 1 to 9 (α-syn: Hsp), and each titration sample was made to a total volume of 500 μL containing of 25 μM ^15^N labeled α-syn in the absence and presence of unlabeled Hsps that diluted from high concentration stocks. Chemical shift deviations (CSD, Δδ) were calculated using equation,

$$\Delta \,\delta =\sqrt{{\left(\Delta \,\delta \,1\,\text{H}\right)}^{2}+0.0289\,{\left(\Delta \,\delta \,15\,\text{N}\right)}^{2}}$$

Where Δδ1H and Δδ15N are the chemical shift differences of amide proton and amide nitrogen between free and bound state of α-syn, respectively. All NMR spectra were processed using NMRPipe (Delaglio et al., 1995) and analyzed using NMRView (Johnson, 2004).

## Results

### Hsp27 Binds to the N-Terminal of α-syn

**FIGURE 1:**
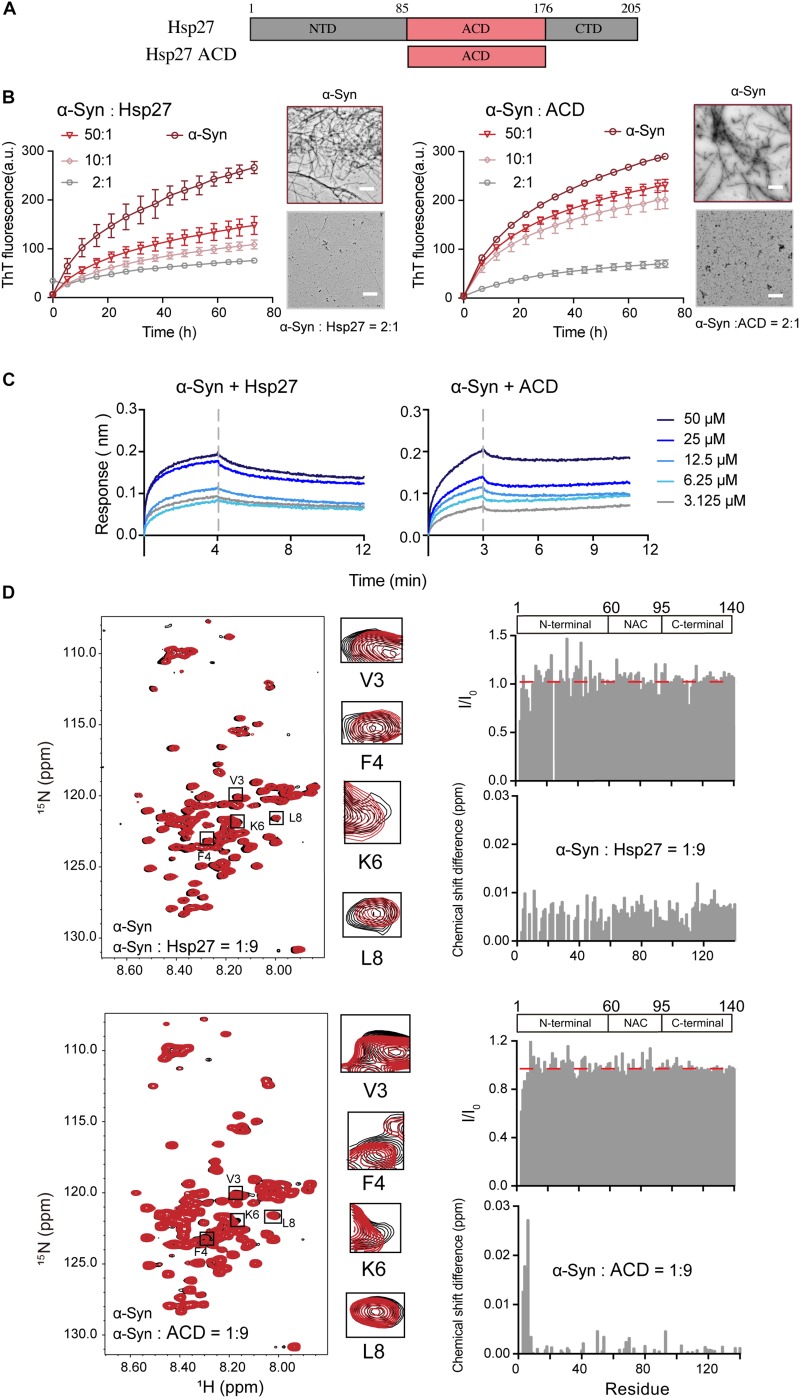
Hsp27 efficiently inhibits α-syn fibril formation by binding to the very N-terminal of α-syn. **(A)** Domain architecture of Hsp27. The central α-crystallin domain (ACD) is flanked by a flexible N-terminal domain (NTD) and a flexible C-terminal domain (CTD). **(B)** ThT kinetics of α-syn (50 μM) aggregation inhibited by Hsp27 (left) and ACD of Hsp27 (right) at the indicated molar ratios, respectively. Error bars correspond to mean ± SD, with *n* = 3. The NS-EM images of α-syn fibrils in the absence and presence of Hsp27/ACD at a molar ratio of 2:1 are shown on the right of the THT curves. Scale bar, 1 μm. **(C)** BLI binding kinetics curves of α-syn with Hsp27 (left) and ACD of Hsp27 (right) at the indicated concentrations, respectively. The association and dissociation profiles are divided by a vertical dash line. The resulting equilibrium dissociation constants (*K*_D_) are 0.2 and 2.2 mM for Hsp27 and ACD to α-syn, respectively. **(D)** Overlay of the 2D ^1^H-^15^N HSQC spectra of 25 μM α-syn in the absence (black) and presence of Hsp27 (top)/ACD (bottom) at a molar ratio of 1:9 (red), respectively. Resonances with relatively large CSD or intensity reduction highlighted in the black boxes are zoomed-in. Residue specific CSDs and intensity ratio (I/I_0_) of α-syn upon titrations are shown on the right. The domain organization of α-syn is shown on the top.

We first sought to investigate whether and how the ubiquitous sHsp — Hsp27 (Figure 1A) modulates the pathological amyloid aggregation of α-syn. α-Syn monomer and Hsp27 multimer were purified from *E. coli* and characterized by gel filtration (Supplementary Figures S1, S2). Consistent with the previous studies, Hsp27 features a high ordered multimer in solution (Lelj-Garolla and Mauk, 2006; Benesch et al., 2008; Jehle et al., 2010). Then, we assessed the chaperone activity of Hsp27 on α-syn aggregation by combining the ThT fluorescence kinetics assay and NS-EM. As shown in Figure 1B, Hsp27 can inhibit amyloid aggregation of α-syn even at a molar ratio as low as 50:1 (α-syn/Hsp27), demonstrating that Hsp27 exhibits potent chaperone activity for α-syn. We further prepared the purified ACD of Hps27 (Figure 1A and Supplementary Figure S2), which was previously reported to be responsible for the chaperone activity of Hsp27 for its amyloid client – Tau (Freilich et al., 2018b). Both the ThT and NS-EM results showed that ACD can prevent α-syn aggregation (Figure 1B and Supplementary Figure S3), while the negative control BSA showed no influence at the same conditions (Supplementary Figure S4), implying that Hsp27 utilizes its ACD to tackle α-syn and inhibit its aggregation.

Next, we sought to investigate the molecular basis underlying the interplay between Hsp27 and α-syn. We firstly measured the binding affinity between Hsp27 and α-syn using the BLI. The equilibrium dissociation constant (*K*_D_) of Hsp27 to α-syn was 0.2 mM (Figure 1C), suggesting that the interaction between them is relatively weak and transient, which is in consist with the previous reports of the weak binding between the conventional chaperones and clients (Baughman et al., 2018). The ACD of Hsp27 binds to α-syn with a slightly weaker binding affinity (*K*_D_) of 2.2 mM (Figure 1C). These results imply that although ACD is sufficient to inhibit α-syn aggregation, the presence of NTD and CTD of Hsp27 may somehow contribute to the binding between them and thus enhance the chaperone activity of full length Hsp27.

Then, we performed solution NMR experiment to identify the interface of α-syn for Hsp27 binding. We prepared the ^15^N-labeled α-syn monomer and collected the two-dimensional (2D) ^1^H-^15^N heteronuclear single quantum coherence (HSQC) spectrum which provides a fingerprint of cross-peaks for each individual non-proline residue. The interface of α-syn can be mapped by recording the changes in the position and/or intensity of each cross-peak upon addition of the binding partner of α-syn. Titration of 9-fold excess unlabelled Hsp27 to ^15^N-labeled α-syn didn’t cause significant changes in chemical shift deviations (CSDs) of the residues of α-syn (Figure 1D). However, the N-terminal residues containing V3-K10 exhibit a relative larger signal reduction of the peak intensities. The signal reduction in the NMR titrations is commonly a combination of two effects: (1) the signal broadening through enhanced transverse relaxation caused by the significantly increased molecular mass upon the transient formation of α-syn-Hsp27 complex, and (2) the chemical exchange at the contact interface. Thus, the NMR titration results indicate that Hsp27 directly binds to the very N-terminal of α-syn.

Then we titrated ACD of Hsp27 to the ^15^N labeled α-syn. Similar to that of the FL-Hsp27, the residues V3-K6 in the N-terminal of α-syn harbor the most significant CSDs (>0.01 ppm) and intensity reduction upon ACD titration (Figure 1D), implying that both ACD and FL-Hsp27 bind to the very N-terminal of α-syn. Of note, ACD features a dimer in solution with MW of 30.0 kDa, which is much smaller than that of full length Hsp27 multimer. Therefore, the binding of ACD to the N-terminal of α-syn results in both CSD and intensity drop rather than the only intensity drop observed in full length Hsp27 binding. Together, our data demonstrates that Hsp27 transiently binds to the very N-terminal of α-syn mainly by its ACD.

### Hsp40 Captures α-syn by Binding to Its C-Terminal

**FIGURE 2:**
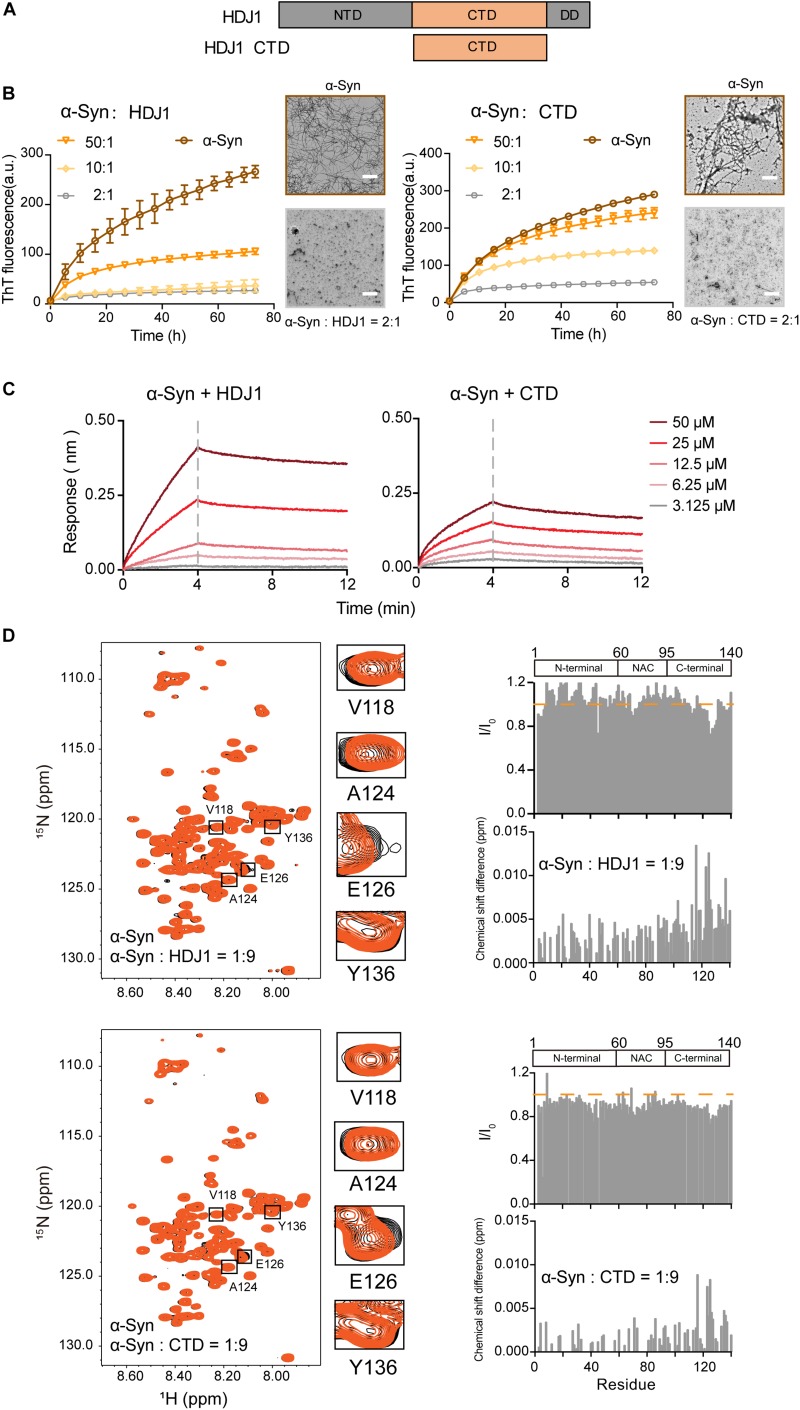
HDJ1 using its CTD to capture the C-terminal of α-syn to prevent its amyloid aggregation. **(A)** Domain architecture of HDJ1, which is composed of an N-terminal domain (NTD), a C-terminal domain (CTD) and a dimerization domain (DD). **(B)** ThT kinetics of α-syn (50 μM) aggregation inhibited by HDJ1 (left) and CTD of HDJ1 (right) at the indicated molar ratios, respectively. Error bars correspond to mean ± SD, with *n* = 3. The NS-EM images of α-syn fibrils in the absence and presence of HDJ1/CTD at a molar ratio of 2:1 are shown on the right of the THT kinetic curves. Scale bar, 1 μm. **(C)** BLI binding kinetics curves of α-syn with HDJ1 (left) and CTD of HDJ1 (right) at the indicated concentrations, respectively. The association and dissociation profiles are divided by a vertical dash line. The resulting equilibrium dissociation constants (*K*_D_) are 1.8 and 3.8 mM for HDJ1 and CTD to α-syn, respectively. **(D)** Overlay of the 2D ^1^H-^15^N HSQC spectra of 25 μM α-syn in the absence (black) and presence of HDJ1 (top)/CTD (bottom) at a molar ratio of 1:9 (orange), respectively. Resonances with relatively large CSD or intensity reduction highlighted in the black boxes are zoomed-in. Residue specific CSDs and intensity ratio (I/I_0_) of α-syn upon titrations are shown on the right. The domain organization of α-syn is shown on the top.

Next, we examined another Hsp — HDJ1 from Hsp40 family (Figure 2A), which acts as a holdase for its chaperone activity on α-syn *in vitro*. As shown in Figure 2B, HDJ1 can efficiently prevent amyloid aggregation of α-syn in a concentration-dependent manner monitored by the ThT kinetics assay and NS-EM. Moreover, CTD of Hsp40, which was previously identified for HDJ1 client recognition (Zarouchlioti et al., 2018), exhibits potent chaperone activity in preventing α-syn amyloid aggregation (Figure 2B). Consistently, the CTD binds to α-syn with a similar binding affinity (*K*_D_: 1.7 mM) to that of full length HDJ1 (3.8 mM) measured by BLI (Figure 2C). These data demonstrate that the CTD of Hsp40 is efficient to interact with α-syn and protect it from aggregation. The high *K*_D_ value of HDJ1 to α-syn further suggests that the interaction between them is weak and transient, and is ∼10-fold weaker than that of Hsp27 to α-syn.

We further performed the NMR titration experiment to pinpoint the interface of α-syn for HDJ1 binding. Titration of 9-fold unlabelled HDJ1 to ^15^N-labeled α-syn resulted in relative larger CSD (>0.006 ppm) of several residues clustered at the C-terminal of α-syn including residues 113, 116, 123–125, 127, 132, 133, and 137 (Figure 2D). In addition, obvious signal reduction was observed around residues 124–130 of α-syn, which is likely caused by the direct binding to the dimeric HDJ1 with MW of 79.4 kDa. NMR titrations were also performed by addition of the CTD of HDJ1 to α-syn. Similar to that of the full-length HDJ1, titration of the CTD of HDJ1 results in obvious CSDs (>0.004 ppm) within the C-terminal of α-syn, e.g., residues of 116, 123–126, 135, and 136. The relative larger signal reduction was also observed around residues 125–130. Taken together, our results suggest that HDJ1 exhibits potential chaperone activity for inhibiting α-syn aggregation by utilizing its CTD to transiently interacting with the C-terminal of α-syn. Notably, despite that both HDJ1 and Hsp27 can bind α-syn, they recognize distinct regions of α-syn monomer.

### Hsp104 Prevents α-syn Aggregation by Binding to Its N-Terminal

**FIGURE 3:**
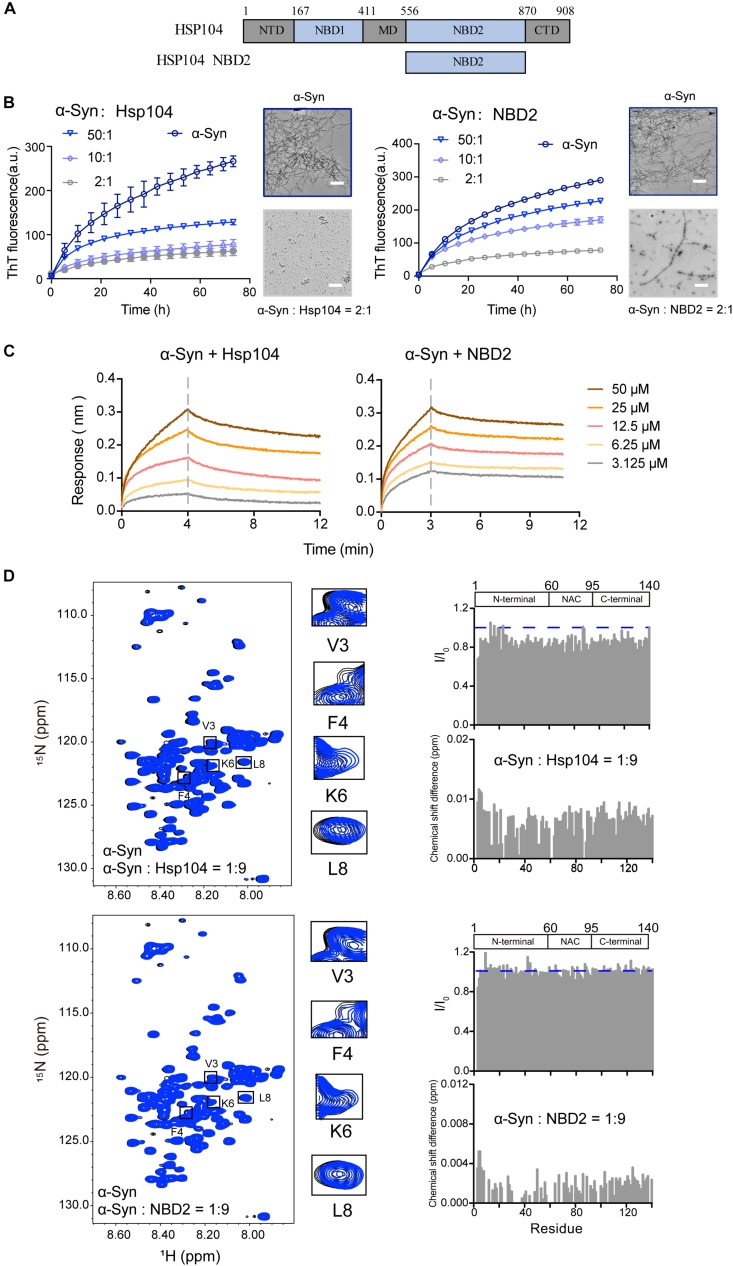
Hsp104 acts as a holdase to inhibit α-syn fibril formation. **(A)** Domain architecture of Hsp104, which consists of 5 domains: N-terminal domain (NTD), nucleotide-binding domains 1 (NBD1), middle domain (MD), NBD2, and C-terminal domain (CTD). **(B)** ThT kinetics of α-syn (50 μM) aggregation inhibited by Hsp104 (left) and NBD2 of Hsp104 (right) at the indicated molar ratios, respectively. Error bars correspond to mean ± SD, with *n* = 3. The NS-EM images of α-syn fibrils in the absence and presence of Hsp104/NBD2 at a molar ratio of 2:1 were shown on the right of the THT kinetic curves. Scale bar, 1 μm. **(C)** BLI binding kinetics curves of α-syn with Hsp104 (left) and NBD2 (right) at the indicated concentrations, respectively. The association and dissociation profiles are divided by a vertical dash line. The resulting equilibrium dissociation constants (*K*_D_) are 1.8 and 3.8 mM for Hsp104 and NBD2 to α-syn, respectively. **(D)** Overlay of the 2D ^1^H-^15^N HSQC spectra of 25 μM α-syn in the absence (black) and presence of Hsp104 (top)/NBD2 (bottom) at a molar ratio of 1:9 (blue), respectively. Resonances with relatively large CSD or intensity reduction that highlighted in the black boxes are zoomed-in. Residue specific CSDs and intensity ratio (I/I_0_) of α-syn upon titrations are shown on the right. The domain organization of α-syn is shown on the top.

We examined a third Hsp – Hsp104 (Figure 3A), which belongs to Hsp100 family and features potent disaggregase activity in dissolving amyloid aggregation formed by different amyloid proteins (e.g., Tau, α-syn) (DeSantis et al., 2012). Recently, we found that Hsp104 can act as a holdcase by utilizing its NBD2 to capture soluble K19 of Tau and prevent it from amyloid aggregation (Zhang et al., 2019). Here, we constructed and purified both FL-Hsp104 and the NBD2 of Hsp104 (Supplementary Figure S2), and studied their interplay with α-syn. As shown in Figure 3B, both Hsp104 and NBD2 were able to effectively inhibit the fibril formation of α-syn in an ATP-independent manner. The results suggested that Hsp104 can function as a holdase for chaperoning α-syn, and the NBD2 is sufficient for this holdase activity. BLI analysis further revealed that the NBD2 binds to α-syn with the binding affinity (*K*_D_) of 0.3 mM (Figure 3C), which is very similar to that of the full length Hsp104 to α-syn (0.4 mM). The binding affinity is relatively low, implying the weak and transient interaction between α-syn and Hsp104.

To map the interface of α-syn for Hsp104 binding, we titrated Hsp104 to ^15^N labeled α-syn, which resulted in the relative larger CSDs and signal reduction at the N-terminal of α-syn including residues of 3, 4, and 6 (Figure 3D), indicating that Hsp104 mainly interacts with the very N-terminal of α-syn. In addition, titration of Hsp104 resulted in a global signal reduction (∼80–90%) across the entire α-syn except some residues, e.g., 13, 14, 16,18–20, 23, 25, 26, 43, 78, 87, 88, 101, 112, 118, 123, and 140, implying much weaker and transient interaction between Hsp104 and the whole α-syn molecule. This is not observed in the titration of Hsp27 to α-syn (Figure 1D), implying that although Hsp104 and Hsp27 both bind to the very N-terminal of α-syn weakly and transiently, their binding patterns are still not the same. In addition, the NBD2 also binds to the similar region in the N-terminal of α-syn (Figure 3D). Together, the results suggest that Hsp104 employs its NBD2 to mainly bind the N-terminal of α-syn for holding it from amyloid aggregation.

### Three Hsps Synergistically Prevent α-syn Aggregation

**FIGURE 4:**
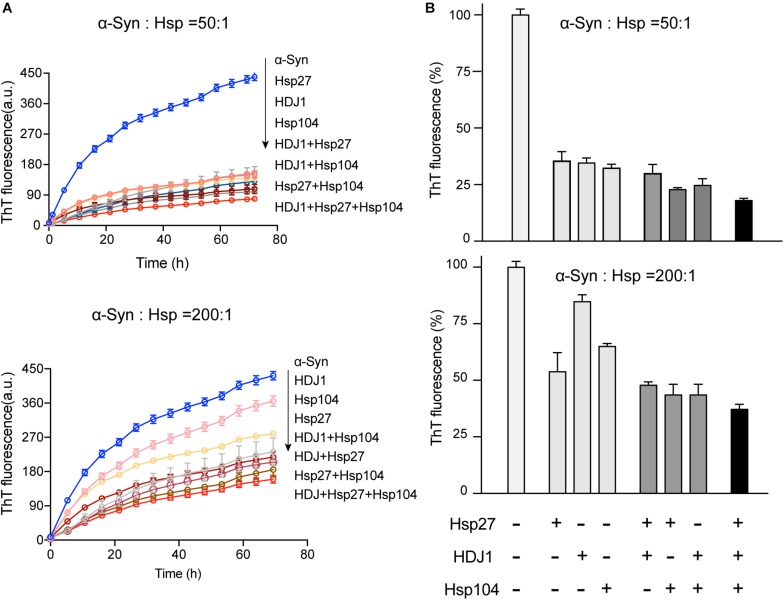
The three Hsps synergistically prevent α-syn amyloid aggregation. **(A)** ThT kinetics of α-syn (50 μM) aggregation inhibited by single Hsp alone (Hsp27, HDJ1, and Hsp104), three binary systems containing equal molar of two Hsps (Hsp27-HDJ1, Hsp104-HDJ1, and Hsp27-Hsp104), and the ternary system (Hsp27: HDJ1: Hsp104 = 1:1:1), respectively. The molar ratios of Hsp to α-syn are 50:1 (top) and 200:1 (bottom), respectively. The total amount of combined Hsps in each assay is equal to that of single Hsp. Error bars correspond to mean ± SD, with *n* = 3. **(B)** Comparison of the chaperone activity of each Hsp alone, the three binary systems and the ternary system for preventing aggregation of α-syn (50 μM). The ThT value was taken at the 72 h time point from the ThT kinetics curves from panel **A**. Error bars correspond to mean ± SD, with *n* = 3. The statistical significance is summarized and listed in the Supplementary Figure S5C.

Intriguingly, our results showed that the three different Hsps bind to distinct segments of α-syn monomer. Therefore, we next ask whether these three Hsps can work synergistically for chaperoning α-syn from aggregation. We firstly compared the binary system containing equal molar of two different Hsps to each of the single Hsp for the chaperone activity on α-syn aggregation. Notably, at low molar ratios of Hsp to α-syn (1:50 and 1:200), each of the three binary systems exhibits significantly higher chaperone activity than that of each individual Hsp alone (Figures 4A,B and Supplementary Figure S5), suggesting that the Hsps indeed prevent α-syn aggregation in a synergistic manner. Of note, when the molar ratio of Hsp to α-syn increases (from 1:50 to 1:10), the binary system of Hsp27-Hsp104 still shows synergy (Supplementary Figure S5). However, the synergistic effect of the binary systems of Hsp27-HDJ1 and Hsp104-HDJ1 dramatically diminishes (Supplementary Figure S5), probably due to saturation of HDJ1 activity at this high ratio of Hsp to α-syn.

Further, we premixed the three Hsps together with equal molar ratio, and examined the chaperone activity of this ternary system. Strikingly, the ternary system exhibits largely enhanced chaperone activity against α-syn aggregation compared to those of either the binary or single-component system (Figure 4 and Supplementary Figure S5). Taken together, these results show that the three Hsps can act synergistically in preventing α-syn aggregation even at an ultra-low molar ratio of Hsps to α-syn (e.g., 1:200).

## Discussion

In this study, we investigated the interaction between α-syn and three Hsps from different families including (1) Hsp27, which is a ubiquitous sHsp; (2) HDJ1 from Hsp40 family serving as a co-chaperone for Hsp70; (3) Hsp104, a well-known disaggregase in the Hsp100 family. Intriguingly, we found that all of these three Hsps can act as a holdase to interact with α-syn monomer via weak and transient interaction, and more importantly, efficiently protect α-syn from amyloid aggregation. These results imply that cell may employ multiple chaperones rather than a single dedicated chaperone in maintaining endogenous α-syn from pathological aggregation.

Moreover, we found that different Hsps recognize distinct regions of α-syn monomer to fulfill their chaperone activities, in which Hsp27 and Hsp104 mainly interact with the very N-terminal of α-syn, while HDJ1 binds to the C-terminal of α-syn. By weakly and transiently binding to the distinct regions of α-syn, the three Hsps may form a chaperone network and work synergistically rather than competitively for preventing α-syn aggregation. The synergistic effect dramatically lowers the effective concentration of chaperones mixture for preventing α-syn aggregation. The premixed ternary system can effectively prevent α-syn aggregation even at a molar ratio of Hsps to α-syn as low as 1 to 200.

**FIGURE 5:**
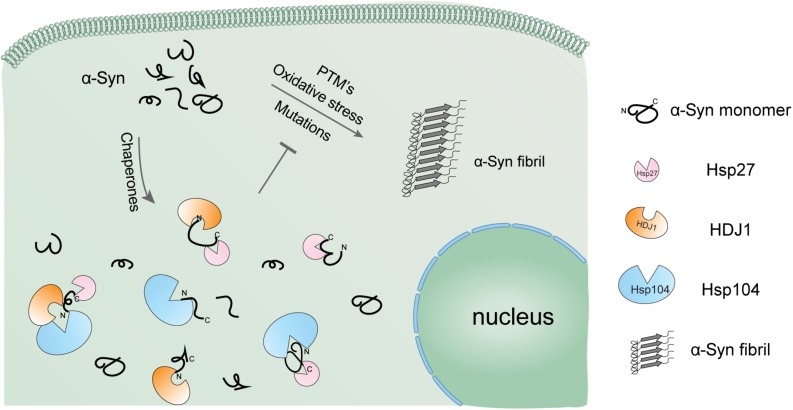
Schematic diagram of the regulation of α-syn aggregation by a variety of Hsps. α-Syn alone is highly prone to form pathological amyloid fibrils, especially under pathological conditions. The cellular chaperone network employs a variety of Hsps using distinct binding mechanisms to hold α-syn monomer in the soluble state and prevent it from amyloid aggregation in a synergistic manner.

In the cellular environment, the highly aggregation-prone amyloid protein like α-syn co-exists with different kinds of chaperones (Lo Bianco et al., 2008; Bruinsma et al., 2011; Pemberton et al., 2011; Gao et al., 2015). Previous studies of the interplay between chaperones and clients are always focused on one specified chaperone and one client. However, a number of chaperones co-exist with client proteins in cells, and it is important to know how different chaperones work together. Our studies of the inhibition effects of each binary system (two Hsps) and the ternary system (three Hsps) on α-syn aggregation indicate that different Hsps work in a synergistic manner. Thus, multiple chaperones might be cooperatively engaged to capture the aggregation-prone amyloid protein by employing distinct binding mechanisms (Figure 5), and prevent it from pathological aggregation. A variety of modification or truncation on endogenous α-syn may occur in cell. For instance, both phosphorylation at the C-terminal (Serine 129) and the C-terminal truncation were observed in the pathological α-syn aggregates in Lewy bodies. Therefore, further study is need to examine whether different Hsps may play distinct roles in targeting different types of α-syn variants.

Our observations show the synergistic effect of different Hsps against α-syn fibril formation. However, these were all from *in vitro* system with purified α-syn and Hsps. Further studies on the potential synergistically effect of different Hsps in preventing α-syn pathological aggregation in cellular and animal model is needed. Beyond that, a large number of co-factors and crowding agents (e.g., ATP, ions, lipids), exist in the complicated cellular chaperone network, their influence on the modulation of α-syn aggregation should also be carefully examined.

## Data Availability Statement

All datasets generated for this study are included in the article/Supplementary Material.

## Author Contributions

All authors listed have made a substantial, direct and intellectual contribution to the work, and approved it for publication.

## Conflict of Interest

The authors declare that the research was conducted in the absence of any commercial or financial relationships that could be construed as a potential conflict of interest.

## Supplementary Material

FIGURE S1 Q-column purification **(A)** and gel filtration characterization **(B)** of α-syn. The purity of α-syn was assessed by SDS-PAGE shown in the figure.

FIGURE S2 Gel filtration characterization of Hsp27 and ACD of Hsp27 **(A)**, HDJ1 and CTD of HDJ1 **(B)**, Hsp104 and NBD2 of Hsp104 **(C)**, respectively. The purity of Hsps were assessed by SDS-PAGE shown in the figure.

FIGURE S3 The NS-EM images of α-syn fibrils in the presence of different Hsps at molar ratios of 50:1 and 10:1, respectively. Scale bar, 1 μm.

FIGURE S4 The influence of BSA on α-syn amyloid aggregation monitored by ThT fluorescence assay.

FIGURE S5 The synergistic effect of Hsps on α-syn aggregation. **(A)** ThT kinetics of α-syn (50 μM) aggregation inhibited by single Hsp alone (Hsp27, HDJ1, and Hsp104), three binary systems containing equal molar of two Hsps (Hsp27-HDJ1, Hsp104-HDJ1, and Hsp27-Hsp104), and the ternary system (Hsp27: HDJ1: Hsp104 = 1:1:1) at a Hsp/α-syn molar ratio of 10:1. Error bars correspond to mean ± SD, with *n* = 3. **(B)** Comparison of the chaperone activity of each Hsp alone, the three binary systems and the ternary system for preventing aggregation of α-syn (50 μM) at a low Hsp/α-syn molar ratio of 200:1. The ThT value was taken at the 72 h time point from the ThT kinetics curves from A. Error bars correspond to mean ± SEM, with *n* = 3. **(C)** Summary of the statistical significance of the comparison of the chaperone activity of each Hsp alone, the three binary systems and the ternary system for preventing α-syn (50 μM) aggregation at Hsp/α-syn molar ratios of 200:1, 50:1, and 10:1, respectively.
