# Supplementary figures and images for: Different Heat Shock Proteins Bind α-Synuclein With Distinct Mechanisms and Synergistically Prevent Its Amyloid Aggregation
Source: Front Neurosci. 2019 Nov 1;13:1124. doi: 10.3389/fnins.2019.01124 (PMC6842937; doi:10.3389/fnins.2019.01124)

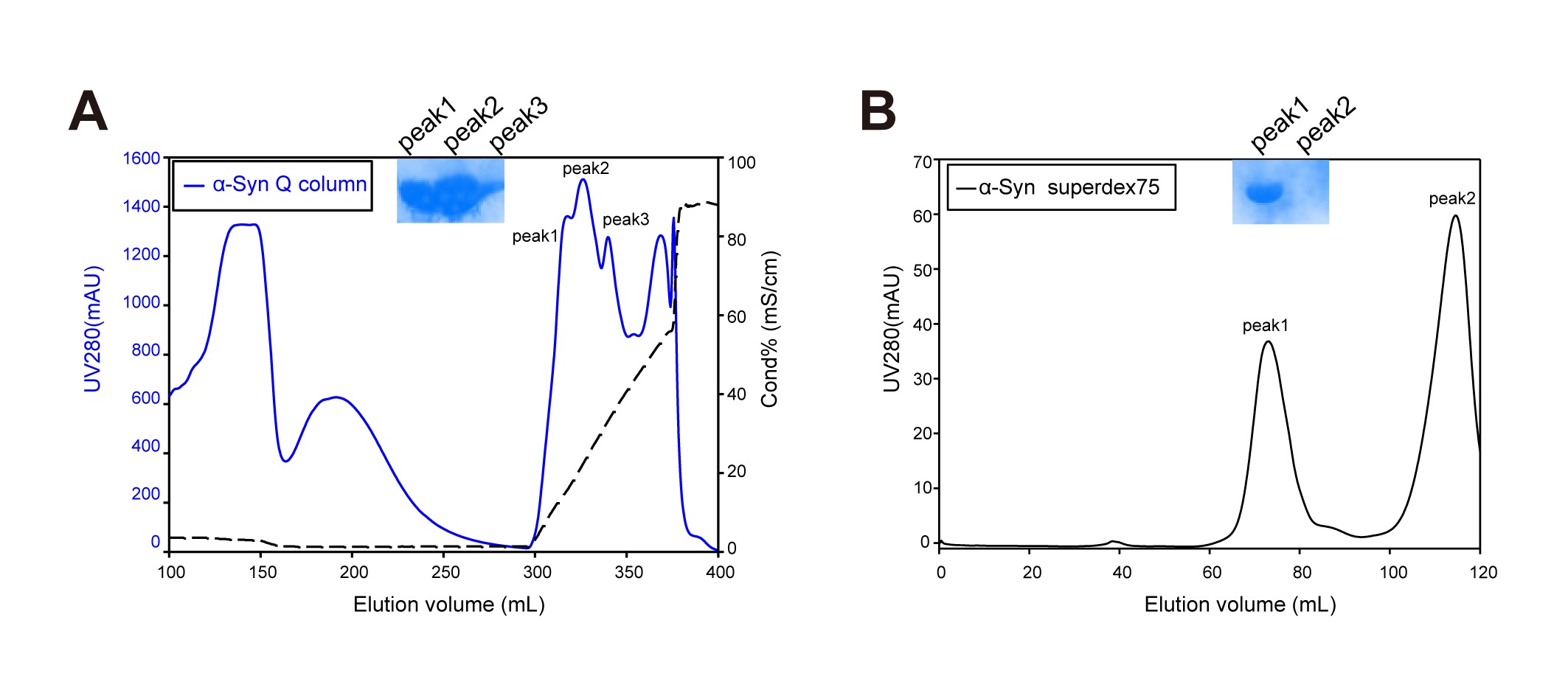

Supplement: FIGURE S1 — Q-column purification (A) and gel filtration characterization (B) of α-syn. The purity of α-syn was assessed by SDS-PAGE shown in the figure. [file Image_1.tif]

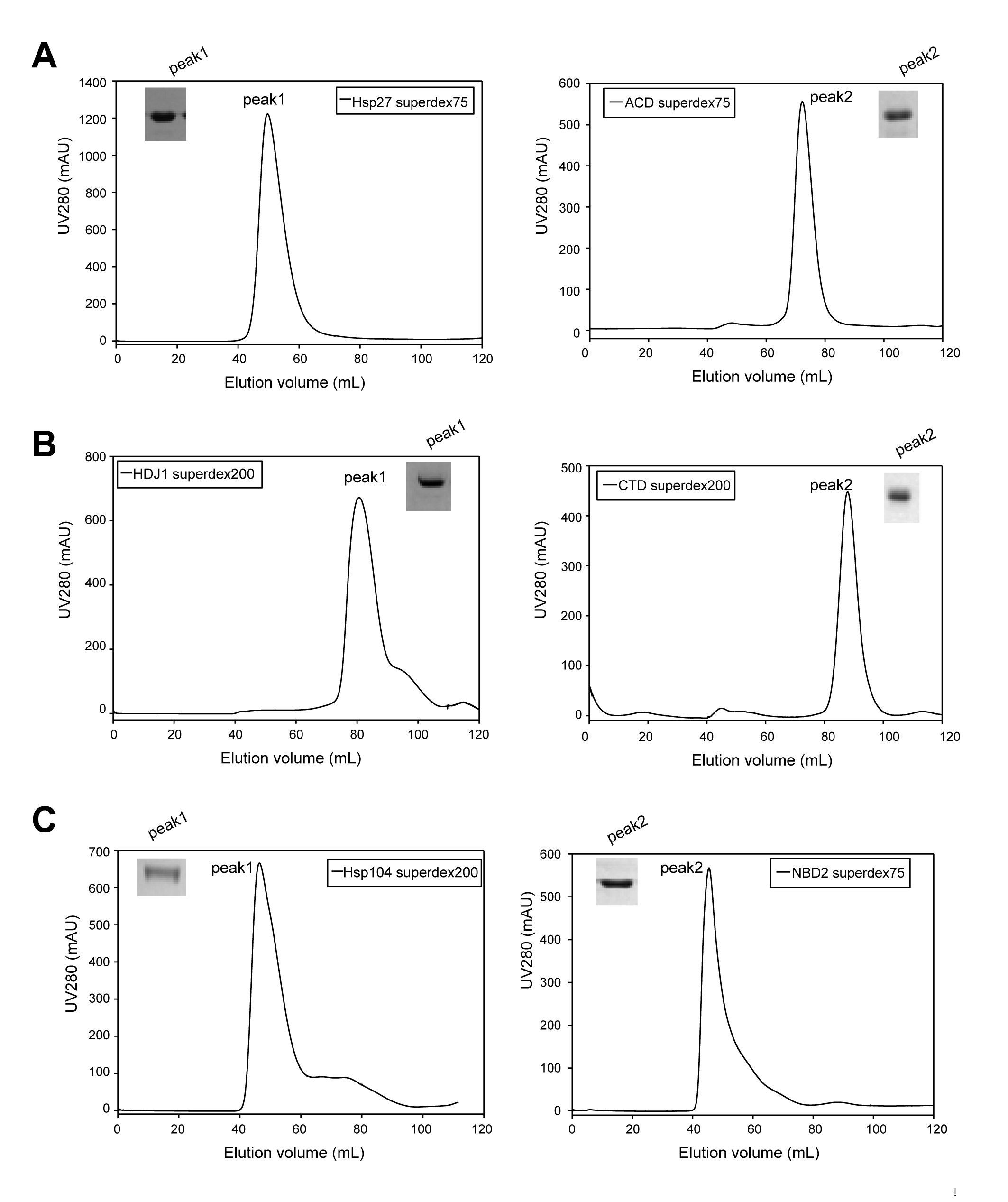

Supplement: FIGURE S2 — Gel filtration characterization of Hsp27 and ACD of Hsp27 (A), HDJ1 and CTD of HDJ1 (B), Hsp104 and NBD2 of Hsp104 (C), respectively. The purity of Hsps were assessed by SDS-PAGE shown in the figure. [file Image_2.TIF]

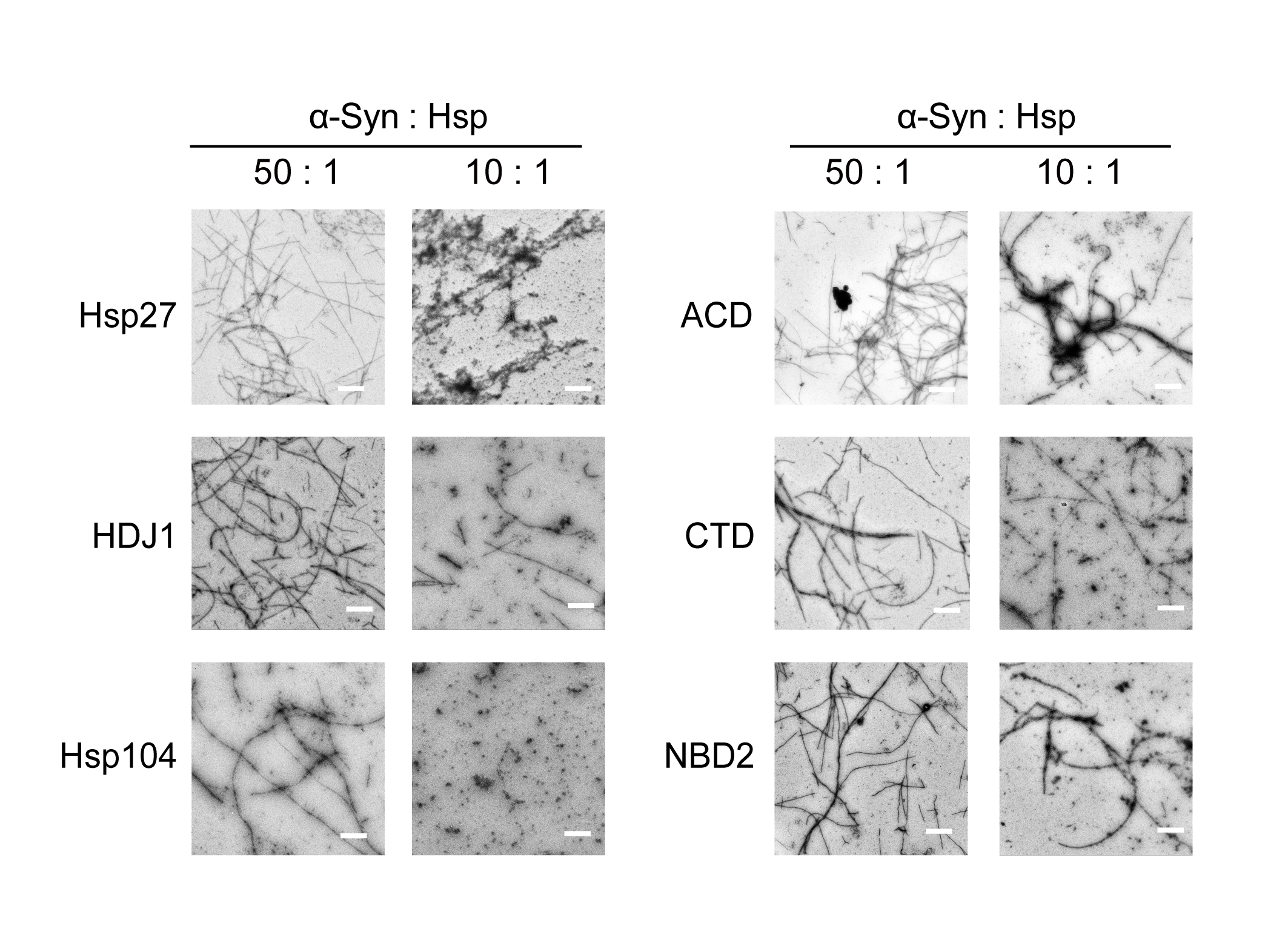

Supplement: FIGURE S3 — The NS-EM images of α-syn fibrils in the presence of different Hsps at molar ratios of 50:1 and 10:1, respectively. Scale bar, 1 μm. [file Image_3.TIF]

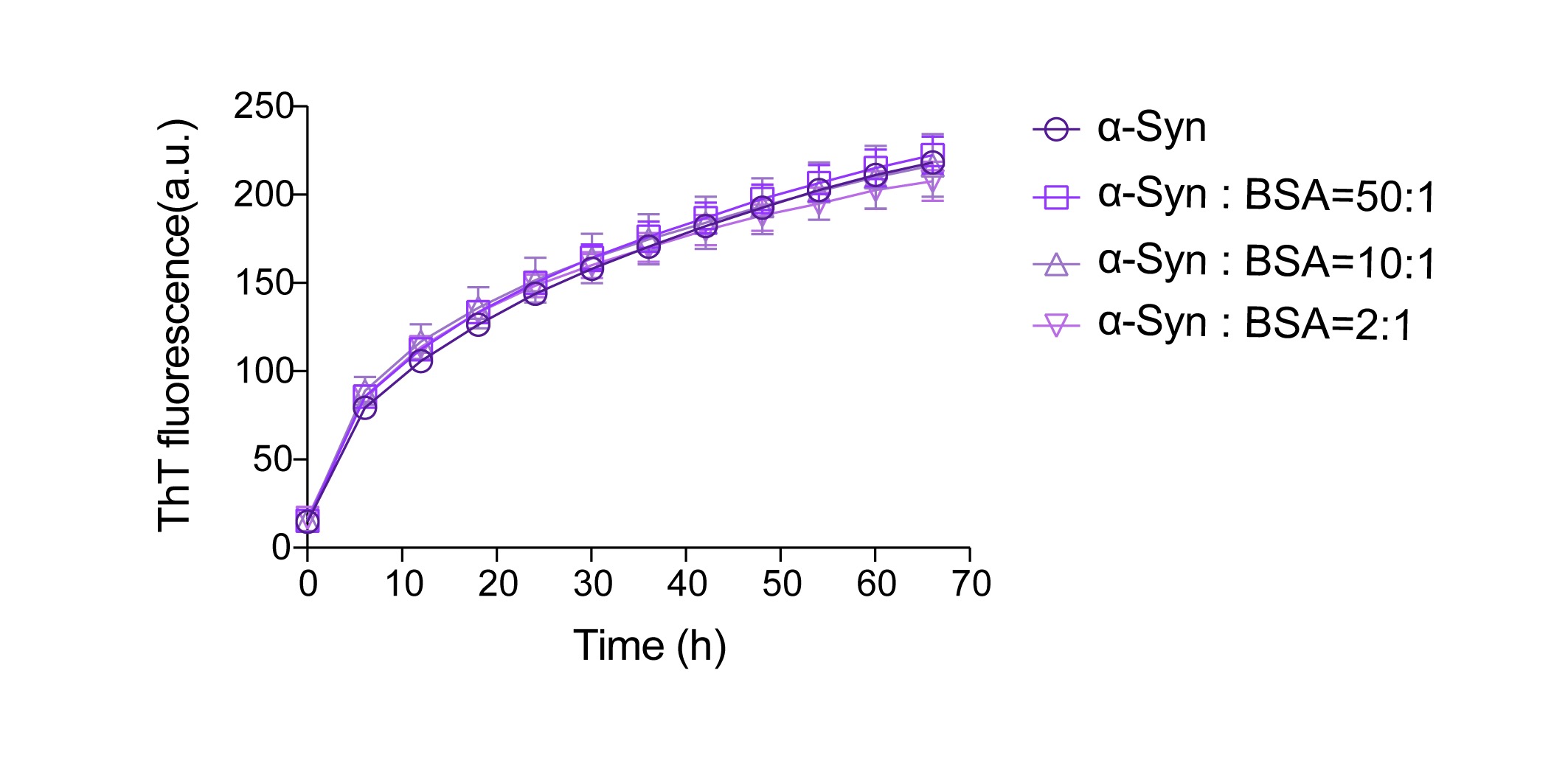

Supplement: FIGURE S4 — The influence of BSA on α-syn amyloid aggregation monitored by ThT fluorescence assay. [file Image_4.TIF]

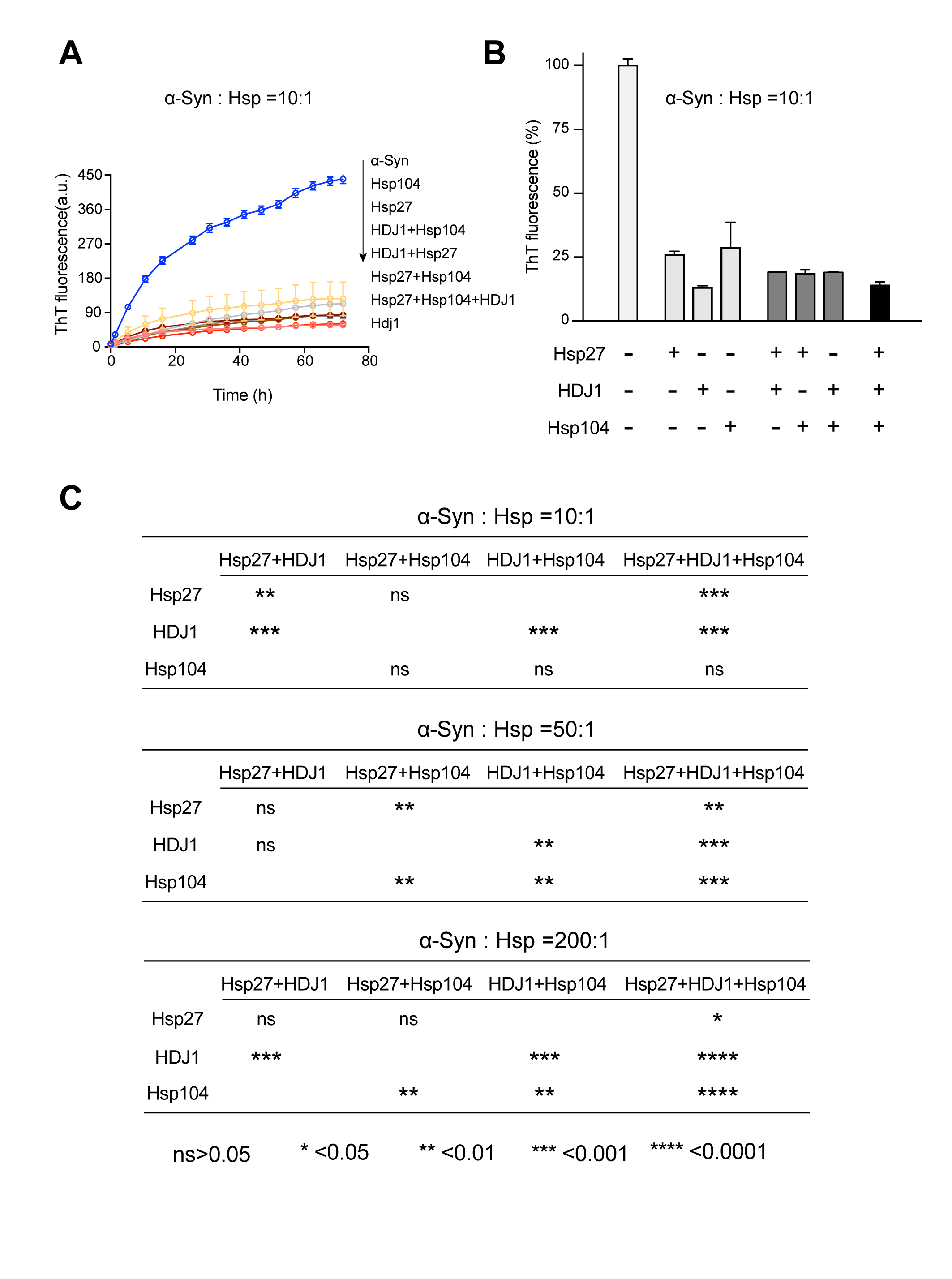

Supplement: FIGURE S5 — The synergistic effect of Hsps on α-syn aggregation. (A) ThT kinetics of α-syn (50 μM) aggregation inhibited by single Hsp alone (Hsp27, HDJ1, and Hsp104), three binary systems containing equal molar of two Hsps (Hsp27-HDJ1, Hsp104-HDJ1, and Hsp27-Hsp104), and the ternary system (Hsp27: HDJ1: Hsp104 = 1:1:1) at a Hsp/α-syn molar ratio of 10:1. Error bars correspond to mean ± SD, with n = 3. (B) Comparison of the chaperone activity of each Hsp alone, the three binary systems and the ternary system for preventing aggregation of α-syn (50 μM) at a low Hsp/α-syn molar ratio of 200:1. The ThT value was taken at the 72 h time point from the ThT kinetics curves from A. Error bars correspond to mean ± SEM, with n = 3. (C) Summary of the statistical significance of the comparison of the chaperone activity of each Hsp alone, the three binary systems and the ternary system for preventing α-syn (50 μM) aggregation at Hsp/α-syn molar ratios of 200:1, 50:1, and 10:1, respectively. [file Image_5.TIF]
